# Self-Esteem Mediates the Relationships Between Social Support, Subjective Well-Being, and Perceived Discrimination in Chinese People With Physical Disability

**DOI:** 10.3389/fpsyg.2019.02230

**Published:** 2019-10-11

**Authors:** Yinyin Ji, Chandni Rana, Congying Shi, Yuan Zhong

**Affiliations:** ^1^School of Psychology, Nanjing Normal University, Nanjing, China; ^2^Honor College, Nanjing Normal University, Nanjing, China; ^3^Department of Biomedical Engineering, University of Florida, Gainesville, FL, United States

**Keywords:** social support, self-esteem, perceived discrimination, subjective well-being, disability

## Abstract

**Background:** Disabilities have a negative effect on mental health and individuals with such conditions are especially susceptible to mental disorders. Previous research has established that in normal population, social support is significantly correlated with subjective well-being (SWB) through mediating variables; however the internal mechanism underlying this in people with physical disability remains unclear. This study aims to examine whether self-esteem and perceived discrimination play a mediating role in the relationship between social support and SWB in a sample of people with physical disability in China.

**Methods:** A total of 210 people with physical disability of Chinese ethnicity were recruited to complete a series of questionnaires. This included the Chinese Social Support Rating Scale (CSSRS), Rosenberg Self-esteem Scale (RSES), Disability Discrimination Perception Questionnaire (DDPQ), and the Chinese Happiness Inventory (CHI). Path analysis was implemented on the data.

**Results:** The model showed excellent fit to data: *χ*^2^ = 2.314, *p* > 0.05; root-mean-square error of approximation (RMSEA) = 0.079; standardized root-mean residual (SRMR) = 0.035; comparative fit index (CFI) = 0.989; and Tucker-Lewis index (TLI) = 0.936. The results showed that self-esteem significantly mediated the relationship between social support and SWB, and perceived discrimination in people with physical disability. However, there is no mediating effect of perceived discrimination between social support and SWB.

**Conclusion:** These findings demonstrate that self-esteem may be a critical resource in mediating the relationships between social support, SWB, and perceived discrimination in people with physical disability.

## Introduction

A disability is defined as a difficulty in functioning at the body, person, or societal levels arising from one or multiple health conditions, often limiting an individual’s ability to thrive (Leonardi et al., 2006). Disabilities can clearly contribute negatively to one’s mental health (Turner et al., 2006) and people with physical disability are especially susceptible to mental disorders. The Chinese government has recently developed more resources for people with physical disability to carry out daily life activities, such as disabled rehabilitation centers in the community. However, mental health was not directly targeted, which is of utmost importance, especially from the perspective of positive psychology.

Subjective well-being (SWB), a major point of study in this report, is defined as the global experience of positive reactions to one’s life, comprising evaluations of life satisfaction, feelings of pleasantness, and lack of unpleasantness (Diener, 1994). The plethora of existing research on SWB has shown that a number of internal and external factors can influence SWB, such as personality, temperament, and living environment (Diener et al., 1995, 2003). Disability, an unfortunate event, is an influential external factor of SWB and may contribute to large declines in SWB (Lucas, 2007). However, certain individuals can often react positively in the face of disability onset, underscoring that the level of SWB can vary in many cases. Those who have good circumstances and take a “person-first” approach often report more happiness (Dunn et al., 2009). Likewise, according to the dual-risk model, some individuals are more susceptible to stressful events from their environment due to their own vulnerability (Belsky and Pluess, 2009). Taken together, these findings show that both individual and environmental factors can affect the SWB of people with disability.

Studies in the normal group have shown that social support, a critical external factor, is significantly correlated with SWB (Cimarolli and Boerner, 2005; Chu et al., 2010; Brajša-Žganec et al., 2018). Social support is defined as an exchange of resources between at least two individuals perceived by the provider or the recipient as having the intention to enhance the well-being of the recipient (Shumaker and Brownell, 1984). It is also defined as the resources provided by others (Cohen and Syme, 1985). Both main- or direct-effect model and buffering model can explain the positive association between social support and SWB in some aspects (Cohen and Wills, 1985). As for people with physical disability, research has also explained that social support can play an important part in improving mental health (Setareh Forouzan et al., 2013). For example, it can regulate depressed mood (Fitzpatrick et al., 1991). Accordingly, we primarily hypothesize that there is a significant positive correlation between social support and SWB in people with physical disability.

Self-esteem, likewise, is an important individual factor and generally refers to a person’s evaluation of the self (Leary and Baumeister, 2000). Self-esteem has been shown to be a strong predictor of SWB in a number of studies (Simsek, 2013; Zhao et al., 2014). Low self-esteem is related to negative psychological consequences such as anxiety (Pyszczynski et al., 2004) and depression (Brown et al., 1986). In previous studies, self-esteem invariably acted as a mediating variable between social support and SWB, revealing a potential mechanism underlying the relationship between social support and SWB in normal people (Kong et al., 2012, 2015). Moreover, social support can influence the self-esteem of adults with disabilities (Nalavany et al., 2015), and studies have found that there was no significant difference in self-esteem between participants with or without disability (Pérez and Garaigordobil, 2007; Arnold and Chapman, 2010). This motivates us to believe that it is also a mediator between social support and SWB of people with physical disability and we hypothesize that self-esteem would have a mediating effect on the impact of social support on SWB of people with physical disability.

Lastly, perceived discrimination, another important individual factor to be considered, refers to the consequences of the subjective perception that one faces discrimination (Schmitt et al., 2014). Perceived discrimination has a significant negative effect on both mental and physical health (Pascoe and Richman, 2009), and is suggested to be another important predictor of SWB (Avidor et al., 2016). Perceived job discrimination can have a significant negative effect on subjective outcomes such as job satisfaction (Hahn and Wilkins, 2013). In the context of perception of discrimination at work (Pérez-Garín et al., 2018), individuals with physical disabilities may experience a heightened sense of inequity and discrimination in compensation in the workplace (Villanueva-Flores et al., 2015), which may negatively affect their SWB. Social support is also closely related to discrimination perception. For example, experiencing parental support can influence adolescents’ symptoms of stress by reducing the effects of perceived discrimination (Jasinskaja-Lahti et al., 2006). Therefore, we hypothesize that perceived discrimination would also have a mediating effect on the impact of social support on SWB in people with physical disability.

**Figure 1:**
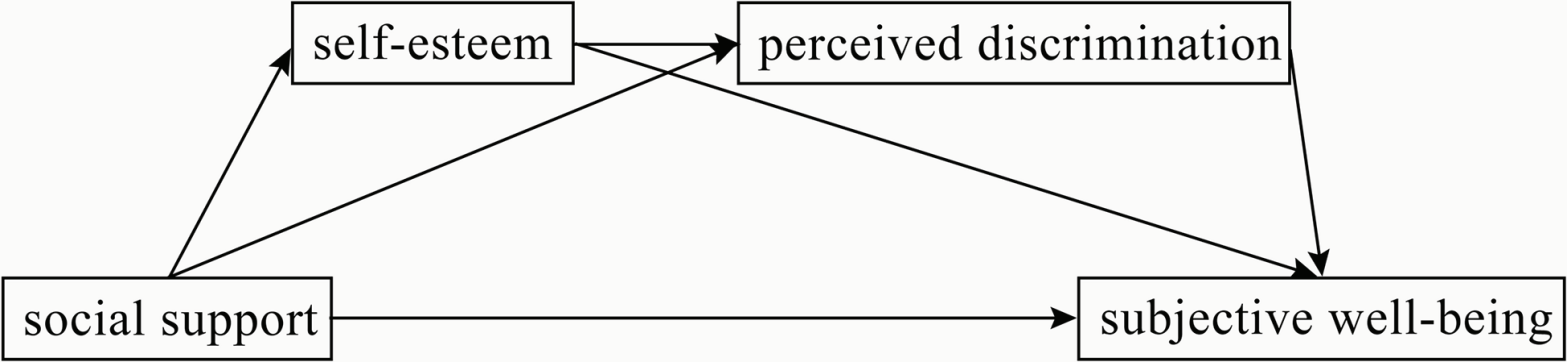
Hypothetical model.

Complex interactions may exist between mediating variables (such as self-esteem and perceived discrimination) that affect SWB, leading to the weakening or even disappearance of a mediating effect. For example, loneliness and self-esteem both play a mediating role between social support and life satisfaction (Kong and You, 2013). Self-esteem may be a significant variable predicting perceived discrimination. Studies have found that perceived discrimination is negatively related to self-esteem (Every and Perry, 2014). Specifically, perceived personal discrimination is related to personal self-esteem, and perceived group discrimination is related to group self-esteem (Verkuyten, 1998). The “self-esteem hypothesis” reveals that individuals’ desire to achieve and maintain positive self-esteem is an important motive in intergroup discrimination (Abrams and Hogg, 1988). Therefore, we further assumed that social support influences SWB through the dual mediators of self-esteem and perceived discrimination. Figure 1 shows our hypothetical model.

In summary, the aim of this study is to examine whether self-esteem and perceived discrimination play a mediating role in the relationship between social support and SWB in a sample of people with physical disability in China. This study has the potential to shed light on the potential psychological mechanism behind this phenomenon and provide much needed insight into possible avenues to address the mental health of people with physical disability.

## Materials and Methods

### Participants

A total of 233 people with physical disability from China were recruited to participate by cluster sampling in several communities in Nanjing. We mainly chose people who had deficiency in walking or missed some of their limbs but still lived a normal life. Questionnaires with more than three missing values were eliminated. Excluding invalid questionnaires, 210 questionnaires were left. The effective rate was 90.13%. Participants ranged in age between 40 and 80 years, of which 110 were male and 100 were female. The mean age of the sample was around 50 years (SD = 9). Ethical approval was obtained from the Ethics Committee of Nanjing Normal University (No. 201907009).

### Measurements

#### Chinese Social Support Rating Scale

The Chinese Social Support Rating Scale (CSSRS) was compiled by Xiao (Xiao, 1986). This scale consisted of 10 items rated on a 4-point Likert scale. It includes three subscales: subjective social support, objective social support, and utilization of social support. Higher scores indicate more social support. For example, “How many close friends do you have to get support and help.” Cronbach’s alpha of the scale was 0.75 in the current study.

#### Rosenberg Self-Esteem Scale

The Rosenberg Self-esteem Scale (RSES) is a 10-item self-report inventory in the Chinese version (Rosenberg, 1965). Participants indicated the extent to which each statement represents their feelings on a 4-point scale from 1 (strongly agree) to 4 (strongly disagree). A higher total score corresponds to a higher level of self-esteem. For example, “On the whole, I’m satisfied with myself.” Cronbach’s alpha of the scale was 0.77 in the current study.

#### Disability Discrimination Perception Questionnaire

The Disability Discrimination Perception Questionnaire (DDPQ) is a 10-item scale to assess the perception of stigma in people with disability (Li, 2013). Items are rated on 5-point scales from 1 (strongly disagree) to 5 (strongly agree). Higher scores are interpreted as higher perception of discrimination. For example, “The tone of the people around me makes me feel unhappy.” Cronbach’s alpha of the scale was 0.90 in the current study.

#### Chinese Happiness Inventory

The Chinese Happiness Inventory (CHI) was compiled by Luluo with a total of 48 items (Lu, 2000). Based on the Oxford Happiness Inventory, the measurement added more items to cover aspects of Chinese happiness. Our study used the very brief version with 10 items, which has been confirmed with good reliability and validity (Lin, 2009; He, 2014). Each item is answered on a 4-point Likert type scale. For example, “I am satisfied with nothing/something/most things/everything in my life.” Cronbach’s alpha of the scale was 0.89 in the current study.

### Procedure

Participants were asked to sign the written informed consent before completing the questionnaire packet consisting of CSSRS, RSES, DDPQ, and CHI. The participants did not place their names on the measures and the confidentiality of their responses was assured. It took approximately 10–15 min for them to complete the packet. Each participant was compensated for his/her participation.

### Data Analysis

Descriptive and correlational analyses were conducted using SPSS 22.0, while mediation analyses were specified using Mplus 7.0. Before conducting analyses, we used One-Sample Kolmogorov-Smirnov Test to check the normality (*p* = 0.2 > 0.05). Pearson correlation analysis was used in the study. The mediation models were estimated using the maximum likelihood estimator. In general, the goodness of fit for each model was assessed by using fit indices including *χ*^2^, CFI, TLI, SRMR, and RMSEA. A non-significant *χ*^2^, values greater than 0.90 for the CFI and TLI, a SRMR less than 0.08, and a value less than 0.08 for the RMSEA are considered to reflect acceptable model fit (Hoyle and Panter, 1995; Hu and Bentler, 1999; Jackson et al., 2009). The statistical significance of mediating and indirect effects was assessed by using bias-corrected bootstrapped estimates of 5,000 bootstrap draws. Statistical significance was determined by 95% bias-corrected bootstrapped confidence intervals that do not contain zero (Efron and Tibshirani, 1993). Harman’s single-factor test was applied to examine common method bias (Podsakoff et al., 2003).

## Results

### Correlational Analysis

The means, standard deviations, and correlation coefficients for all study variables are displayed in Table 1. The results showed that social support was positively correlated with self-esteem and SWB, and negatively correlated with perceived discrimination. Self-esteem was significantly negatively related with perceived discrimination, while significantly positively associated with SWB. Furthermore, perceived discrimination was significantly negatively correlated with SWB.

**Table 1:** Mean, standard deviations (SD), Cronbach’s alpha coefficients, and correlations of all the variables (*N* = 210).

	1	2	3	4
1. Social support	1			
2. Self-esteem	0.34^**^	1		
3. Perceived discrimination	−0.23^**^	−0.42^*^	1	
4. Subjective well-being	0.37^**^	0.43^**^	−0.20^*^	1
*M*	3.83	2.83	2.53	2.42
SD	0.93	0.42	0.77	0.62
*α*	0.75	0.77	0.90	0.89

* *p* < 0.05

** *p* < 0.01.

### Mediation Analysis

The study conducted an exploratory factor analysis for all items relevant to the study. We found that this procedure suggested nine factors, while no single factor accounted for the majority of the covariance among the variables. Moreover, we loaded all the questions of variables on only one common factor, and built a single-factor structural equation model. The model showed undesirable fit to data: *χ*^2^/df = 4.520; RMSEA = 0.129; SRMR = 0.143; CFI = 0.361; and TLI = 0.326. Therefore, no significant common method bias was present in the current study.

**Figure 2:**
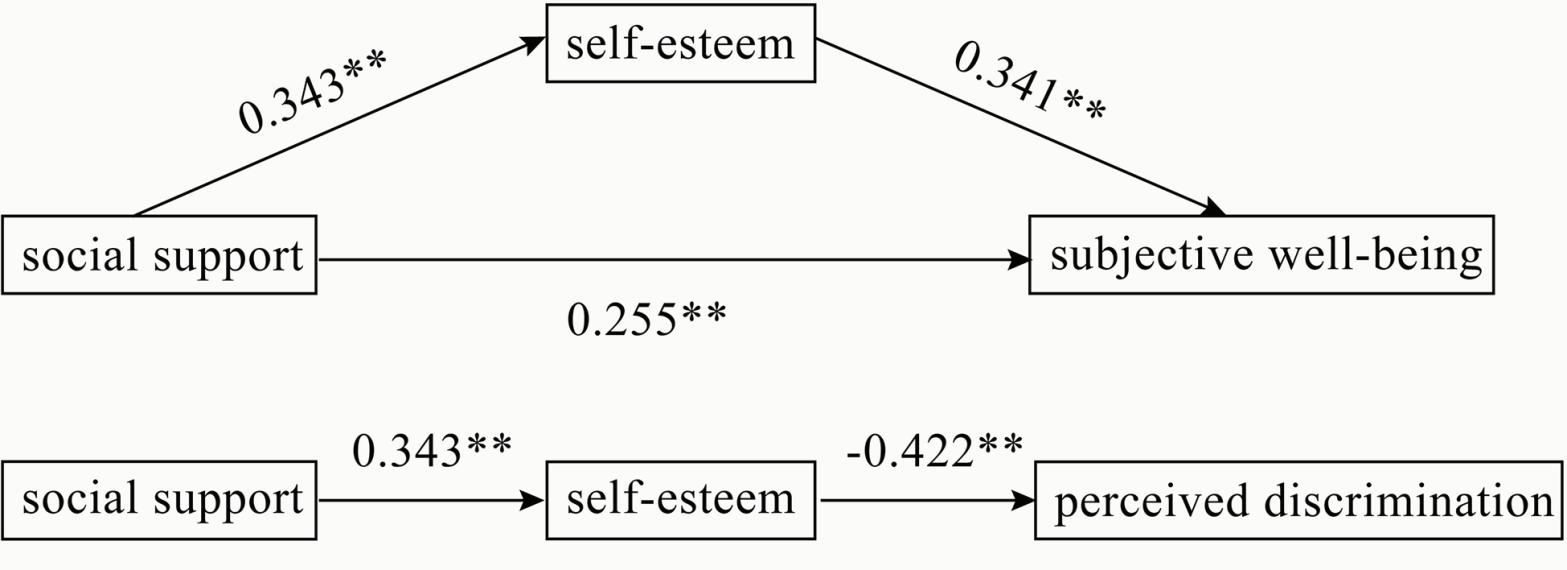
Path coefficients of the model (the coefficients are standardized, ^**^p < 0.01).

We adopted the path analysis method to test the relationship between social support, self-esteem, perceived discrimination, and SWB. The analysis results showed that the beta values of the two paths (social support → perceived discrimination, perceived discrimination → SWB) were − 0.10 and 0.01 respectively (all *p* > 0.05). Therefore, we deleted the two paths and re-analyzed. Figure 2 shows the total effect of social support and the indirect effect through self-esteem. In the model, we found that social support and self-esteem can significantly predict SWB. Social support can also significantly predict self-esteem and perceived discrimination. Moreover, self-esteem played an important mediation role in the relationship of social support to SWB. Meanwhile, it fully mediates the relationship between social support and perceived discrimination. However, there is no mediating effect of perceived discrimination between social support and SWB. The model showed excellent fit to data: *χ*^2^ = 2.314, *p* > 0.05; RMSEA = 0.079; SRMR = 0.035; CFI = 0.989; and TLI = 0.936.

Results showed that self-esteem significantly mediated the relationship between social support and SWB (95% CI = 0.07–0.17), and perceived discrimination (95% CI = −0.22 to −0.07).

## Discussion

The present study investigated the relationship between social support, self-esteem, perceived discrimination, and subjective well-being in people with physical disability. Our study tested four hypotheses: that (1) social support would be related to SWB; (2) self-esteem would mediate the relationship between social support and SWB; (3) perceived discrimination would mediate the relationship between social support and SWB; and (4) social support would influence SWB through the dual mediators of self-esteem and perceived discrimination. As predicted, social support was found to positively predict SWB, supporting previous findings on social support and SWB (Turner, 1981; Parasuraman et al., 2010). Although both the main effect model and the buffering model can explain the relationship of social support and SWB (Cohen and Wills, 1985), for people with physical disability, it seems that the buffering model is more appropriate. Disability is a kind of stress which is closely linked to helplessness, low self-esteem, and other negative emotions. More social support means better buffering effects on stressors, thus producing a positive impact on SWB. Moreover, our study found that self-esteem mediated the relationship between social support and SWB, as well as social support and perceived discrimination in people with physical disability. However, inconsistent with our hypothesis 3, we did not find a mediating effect of perceived discrimination between social support and SWB.

The most significant finding of our study is the mediating effect of self-esteem in two models. For one, self-esteem plays a mediating role between social support and SWB, which has been confirmed in numerous studies (Kong et al., 2013; Tian, 2016). In people with physical disability, we demonstrated this mediating effect once more. The more social support an individual with physical disability receives, the higher his or her self-esteem will be, leading them to attain greater SWB. Additionally, self-esteem is a mediator between social support and perceived discrimination. With greater received social support, the higher their self-esteem will be, and the level of perceived discrimination will be lower. The Dohrenwend model argues that good situational and mental regulation are beneficial coping strategies for people when faced with stress events like disability in their life (Dohrenwend, 1978). It implies that social support (situational regulation) and self-esteem (mental regulation) are potential factors linked with positive consequences such as more SWB and less perceived discrimination.

According to the identity theory of self-esteem, self-esteem is an outcome of, and necessary ingredient in, the self-verification process. Self-esteem buffers the negative emotions that occur when self-verification is problematic (Cast and Burke, 2002), for instance, when faced with a stressor or depression (Hall et al., 1996). Identity consistency is associated with SWB to some extent (Suh, 2002). Consistent with the dual-risk model of individual development, people with a disability tend to have a high degree of sensitivity, which means that they are vulnerable to suffer from external influences. In the case of comparing themselves with others, they are less likely to accept and identify themselves, which easily leads to negative emotions, low life satisfaction, and sensitivity to others’ perspectives. In our study, we provide evidence that social support may elicit positive emotions, higher SWB, and lower perceived discrimination. This may be because individuals with more social support feel a more positive social atmosphere and tend to form the concept of self-respect and self-care, further promoting the level of SWB. Due to the role of self-esteem, it is necessary to provide ample social support, and pay more attention to the establishment of their social networks in order to improve the level of self-esteem of people with disability.

There was no evidence that perceived discrimination was related to SWB, which is slightly different from our initial hypothesis. Previous studies indicate that perceived discrimination often plays a role as a mediator or a moderator (Fuller-Rowell et al., 2012; Coutinho and Blustein, 2013). This may result from the insensitivity of perceived discrimination. Different levels and types of self-esteem can influence the psychological responses to perceived discrimination, and then affect psychological health (Corning, 2002). Meanwhile, perceived discrimination and psychological well-being can be mediated by sense of control (Jang et al., 2008), such as a person’s belief in a just world for self (Schaafsma, 2013). Among literature, self-stigma, resulting from perceived mental illness stigma, is highly related to perceived discrimination (Brohan et al., 2010). And, the association between self-stigma and quality of life is verified among different populations (Lin et al., 2016; Chan et al., 2017; Chang et al., 2019). It is possible that perceived discrimination does not have strong association with SWB because it does not directly link to SWB as the self-stigma does. Therefore, to account for our results, we assume that there seem to exist some mediators or moderators between perceived discrimination and SWB (such as self-stigma), thus weakening the degree of the direct impact.

Several limitations ought to be noted. First, the study has a relatively small sample size and mainly focuses on the middle-aged and elderly. Meanwhile, more demographics such as educational level, family income, health behaviors, and family support were not included. Differences of these variables in different age groups can be discussed in further studies. Second, people with disability only account for a fraction of the total population of people with physical disability. To draw a more general conclusion, further studies should concentrate on other types of disabilities. Finally, the mechanism between social support and SWB is indeed complicated. In this study, we discover the mediating role of self-esteem between social support and SWB. However, there are still some variables that can affect that relationship such as psychological capital, where few studies have focused on people with disability. Furthermore, future research should focus on determining more precisely what influences people with disability physically and psychologically. Meanwhile, we also want to suggest future studies on family members. Family members of people with disability may suffer from high stress, depression, and low quality of life (Cummins, 2001; Michalik, 2015), which can have a negative effect on their mental health. Therefore, more research ought to pay attention to mental problems they may encounter.

To conclude, adequate social support for people with physical disability may improve their self-esteem, thus impacting their perceived discrimination and SWB. Improving social support in families and neighborhoods may alleviate distress and foster hope (Cheng et al., 2014). In order to improve SWB of people with physical disability, we need to build a comprehensive social support network for them, providing them with material and spiritual support. Families of people with physical disability, communities, and social organizations ought to also be involved to create a good living environment to improve the lives of people with physical disability.

## Data Availability Statement

The datasets generated for this study are available on request to the corresponding author.

## Ethics Statement

This study was approved by the Ethics Committee of Nanjing Normal University (No. 201907009), and written informed consent was given by all participants prior to participation.

## Author Contributions

CS and YZ contributed to conception and design of the study. YJ organized the database, performed the statistical analysis, and wrote the first draft of the manuscript. YJ, CR, YZ, and CS contributed to manuscript revision and read and approved the submitted version. CS, as the corresponding author, takes primary responsibility for communication with the journal and editorial office during the submission process, throughout peer review and during publication.

### Conflict of Interest

The authors declare that the research was conducted in the absence of any commercial or financial relationships that could be construed as a potential conflict of interest.
